# Impairment of hypoxia-induced angiogenesis by LDL involves a HIF-centered signaling network linking inflammatory TNFα and angiogenic VEGF

**DOI:** 10.18632/aging.101726

**Published:** 2019-01-18

**Authors:** Fengyan Jin, Xiangyu Zheng, Yanping Yang, Gang Yao, Long Ye, Thorsten R. Doeppner, Dirk M. Hermann, Haifeng Wang, Yun Dai

**Affiliations:** 1 Department of Hematology, Cancer Center, the First Hospital of Jilin University, Changchun, Jilin, China; 2 Department of Neurology, the First Hospital of Jilin University, Changchun, Jilin, China; 3 Department of Neurology, the Second Affiliated Hospital of Jilin University, Changchun, Jilin, China; 4 Laboratory of Cancer Precision Medicine, the First Hospital of Jilin University, Changchun, Jilin, China; 5 Department of Neurology, University Medical Center Göttingen, Göttingen, Germany; 6 Department of Neurology, University Duisburg-Essen Medical School, Essen, Germany; 7 Department of Neurosurgery, the First Hospital of Jilin University, Changchun, Jilin, China

**Keywords:** low-density lipoprotein, hypoxia-inducible factor, tumor necrosis factor-α, vascular endothelial growth factor, nuclear factor-kappaB, angiogenesis

## Abstract

Hypoxia inducible factors (HIFs) mediate angiogenesis via up-regulation of various pro-angiogenic factors (particularly VEGF) in response to hypoxia. Here, we report that hypoxia unexpectedly induced robust production of the pro-inflammatory factor TNFα by endothelial cells (ECs), suggesting an autocrine loop that in turn activated HIFs via an NF-κB-dependent process, resulting in production of VEGF and thereby promotion of angiogenesis. In contrast, low-density lipoprotein (LDL) prevented expression of HIFs in ECs exposed to either hypoxia or TNFα, while knockdown of either HIF-1α or HIF-2α strikingly attenuated hypoxia-induced production of VEGF by ECs as well as EC colony formation and tube formation. Significantly, LDL attenuated hypoxia-induced angiogenesis by disrupting the TNFα/NF-κB/HIF/VEGF signaling cascade via down-regulation of the TNF receptor TNF-R1, rather than TNFα itself, and multiple key components of both canonical and non-canonical NF-κB pathways. By doing so, LDL was able to either inhibit or down-regulate a wide spectrum of HIF-dependent pro-angiogenic downstream targets and signals. Together, these findings argue existence of a self-regulatory TNFα/NF-κB/HIF/VEGF signaling network in ECs, which mediates and fine-tones angiogenesis, at least in response to hypoxia. They also suggest that LDL impairs angiogenesis by disrupting this network, which might represent a novel mechanism underlying anti-angiogenic property of LDL.

## Introduction

Hypoxia-driven angiogenesis represents one of the major adaptive responses to low oxygen supply, which is essential for maintenance of cellular metabolism, survival, and function, thereby representing a prominent feature of embryo development, ischemic injury recovery, tissue regeneration, inflammation, tumor growth, etc [1]. This event is primarily governed by the transcription factor hypoxia-inducible factors (HIFs), including HIF-1α and HIF-2α (originally named endothelial PAS domain protein-1, *EPAS1*), HIF-3α, and HIF-1β (also known as aryl hydrocarbon receptor nuclear translocator, *ARNT*) [2]. Different HIF isoforms may have their distinct functions in regulation of hypoxia-induced metabolic switch in endothelial cells (ECs) and angiogenesis [2,3]. It has been well established that as a major transcription factor, the HIF complex consisting of an oxygen-sensitive subunit (HIF-1α or HIF-2α) and a constitutively expressed HIF-1β plays an essential role in driving angiogenesis under various physiological and pathological conditions [4,5]. In the latter case, the significance of HIF-mediated angiogenesis (via VEGF) has been well documented in a wide variety of disease types, especially aging-related diseases such as atherosclerotic (e.g., coronary artery disease, ischemic stroke) [5] and degenerative disorders (e.g., neurodegeneration [6] and macular degeneration [7]). In diverse types of cells (especially ECs), downstream genes of HIF are also involved in vascular remodeling, inflammation, cell survival and proliferation, apoptosis, autophagy, cell migration and invasion, DNA damage responses, extracellular matrix metabolism, as well as glucose metabolism (e.g., glucose uptake and glycolysis) [8,9].

Among the HIF family members, HIF-1α is ubiquitously expressed in virtually all mammalian tissues and cell types, thereby involved in most, if not all, of cellular responses to hypoxia, particularly including initiation and promotion of angiogenesis via up-regulating a broad spectrum of angiogenic factors (e.g., VEGF, iNOS, HO1) in various types of cells, particularly ECs [10,11]. HIF-2α is however selectively expressed in certain cell types, mainly vascular ECs, and has been recently implicated in regulation of angiogenesis and vascular remodeling by inducing expression of endothelial-specific genes (e.g., VEGF) [12]. Despite ~50% amino acid identity and analogous protein structure, HIF-1α and HIF-2α seem to play distinct, non-redundant biological roles in various physiological and pathological processes, including angiogenesis, likely via activation of different target genes [2,3]. For example, HIF-1α knockout results in severe blood vessel defect [13], while HIF-2α deletion causes defective vascular remodeling during embryonic development [12]. Whereas some genes (e.g., VEGF and other genes that contain HRE) are activated by both HIF-1α and HIF-2α, others are preferentially activated by one or another [14]. As the main sensors of hypoxia, HIF-1α and HIF-2α share a similar mechanism for fast protein turnover, which plays a predominant role in determining their protein levels, although likely depend upon different degrees and durations of oxygen availability [2]. Under normoxic conditions, HIF-1α and HIF-2α are hydroxylated by prolyl-hydroxylase domain (PHD) at the conserved proline residues (HIF-1α, P402 and P564; HIF-2α, P405 and P531) and thus recognized by the von Hippel-Lindau protein (pVHL) complex, an E3 ubiquitin ligase, leading to their ubiquitination and degradation via the ubiquitin-proteasome system (UPS) [15]. In contrast, hypoxia prevents the oxygen-dependent hydroxylation of these two HIF α subunits, which then form active heterodimer complexes with the regulatory subunit HIF-1β, thereby triggering transcription of target genes with diverse functions [15]. Unlike its partners HIF-1α and HIF-2α, HIF-1β expression has long been considered to be constitutively stable and barely affected by hypoxia. In addition to the well-established relationship between NF-κB and HIF-1α [16,17], activation of the canonical NF-κB pathway has also been shown to up-regulate HIF-1β at transcriptional level [17,18]. Moreover, activation of the non-canonical NF-κB pathway (e.g., by TNFSF14/LIGHT) also induces expression of HIFs (particularly HIF-2α) at transcriptional level, as the NF-κB subunit p52 can directly bind to multiple sites on the HIF-2α promoter [19]. These findings suggest an alternative cross-talk between these two major transcription factors involved in various adaptive responses to hypoxia, including angiogenesis.

The functions of HIFs in ECs as well as immune cells (e.g., macrophages) have been well documented, especially in association with inflammation and angiogenesis in atherosclerosis [20]. For example, low shear stress induces HIF-1α expression in ECs likely via an NF-κB-dependent process, an event that enhances EC proliferation and inflammatory activation [21]. Oxidized LDL (oxLDL) activates HIF-1α in macrophages, which transmits pro-angiogenic effects of oxLDL by linking hyperlipidemia, inflammation, and angiogenesis together [22]. TNFα, one of the most important pro-inflammatory cytokines mainly produced by immune cells (e.g., macrophages), induces HIF activation in macrophages during atherogenesis [23]. In the previous study, we have observed that as a classic activator of NF-κB, TNFα might also be involved in activation of the HIF pathway in ECs [18]. However, the role of HIF-1α and/or HIF-2α is unclear in regulation of TNFα-induced angiogenesis, especially under hypoxia.

As a major risk factor, hypercholesterolemia is well known to be associated with atherosclerosis due to depositing lipids into the blood vessel wall. Alternatively, several groups including ours have observed that LDL is able to attenuate angiogenesis, particularly in response to hypoxia [24–27]. We have also demonstrated that the anti-angiogenic activity of LDL involves reduction of both HIF-1α and HIF-2α protein levels in ECs, likely in association with inactivation of NF-κB and resulting down-regulation of HIF-1β [18]. However, it remains unknown whether and how TNFα is involved in hypoxia-mediated angiogenesis in ECs. Here, we report for the first time, to the best of our knowledge, that hypoxia induces production of TNFα by ECs, representing an autocrine loop that in turn activates the HIF pathway via an NF-κB-dependent process, which facilitate VEGF production by ECs and angiogenesis. Therefore, these findings provide evidence suggesting a self-regulatory TNFα/NF-κB/HIF/VEGF singling network in ECs, which mediates angiogenesis at least in response to hypoxia. They also argue that LDL might act to impair hypoxia-induced angiogenesis via disruption of this signaling cascade.

## RESULTS

### The GEP analysis reveals a potential HIF-centered signaling network in ECs

**Figure 1:**
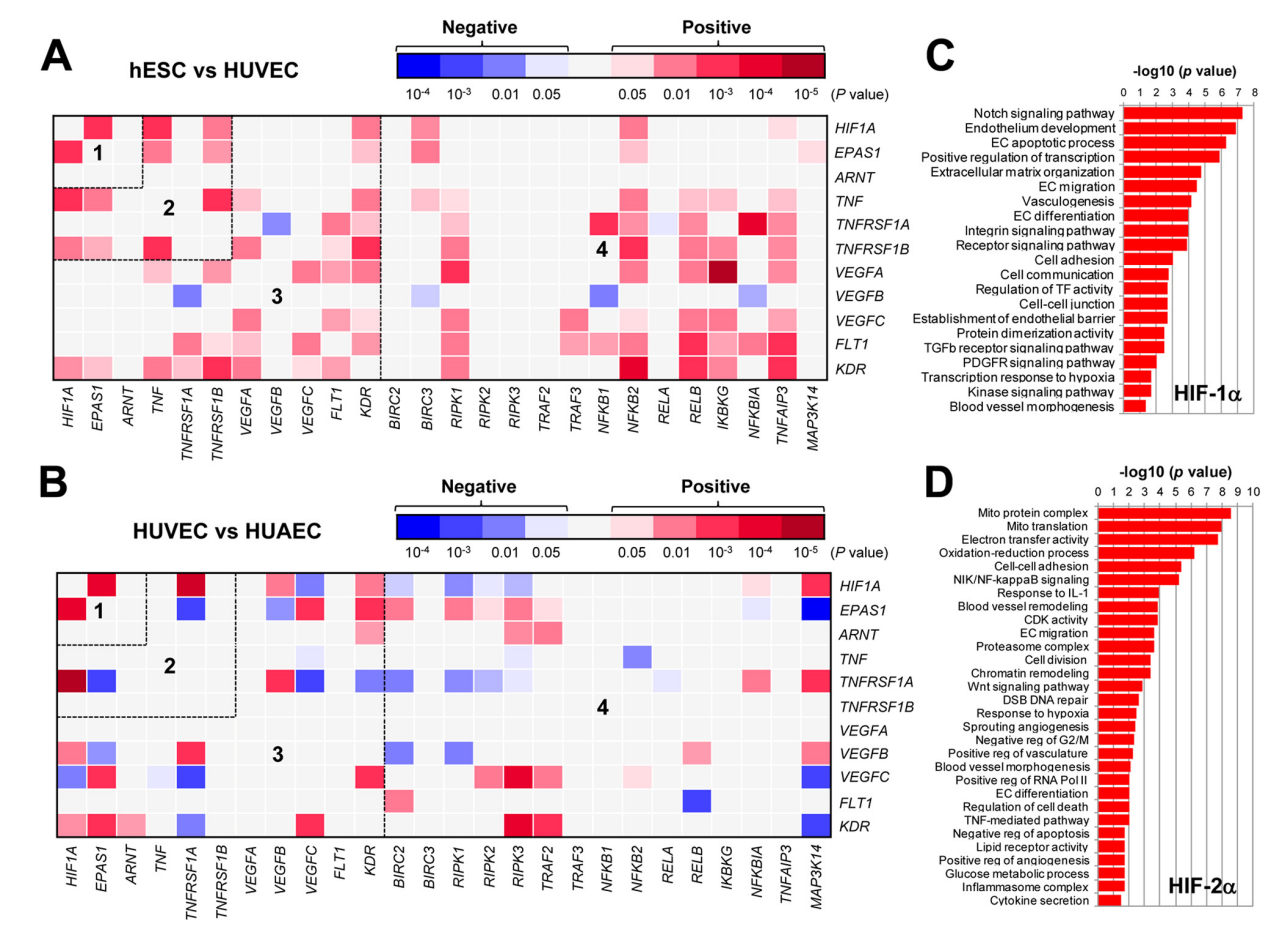
** The GEP analyses map the potential correlations among the HIFs, TNFα, VEGFs, and NF-κB signaling pathways in ECs.** (**A**, **B**) By analyzing the datasets involving gene expression profiling (GEP) in ECs using the R2: Genomics Analysis and Visualization Platform, the heatmaps were generated based on *P* values for analyses of correlations (red and blue indicating positive and negative correlations, respectively) between each pair of genes as indicated on longitudinal and transverse axes, in (**A**; dataset, Exp HUVEC vs ESC - James - 12 - MAS5.0 - u133p2) human stem cells (hESC) vs human umbilical vein endothelial cells (HUVEC) and (**B**; dataset, Normal Endothelial Cells HUAEC/HUVEC - Luttun - 38 - MAS5.0 - u133p2) HUVEC vs human umbilical artery endothelial cells (HUAEC). Numbers in the heatmaps indicate the areas (outlined by dash line) clustered for each pathway. (**C**, **D**) Gene ontology (GO) analyses were performed to categorize (**C**) HIF-1α- and (**D**) HIF-2α-related genes according to their functions, in all types of ECs, including (dataset, Normal Endothelial Cells HUAEC/HUVEC - Luttun - 38 - MAS5.0 - u133p2).

It is well-established that HIFs play an essential role in angiogenesis via inducing expression of pro-angiogenic VEGF as well as its receptors VEGFR1 and VEGFR2 in ECs under both hypoxic and normoxic conditions [2,4]. It is also known that HIFs (HIF-1α in particular) are involved in production of pro-inflammatory TNFα by macrophages, which in turn stimulate angiogenesis or vascular remodeling likely by activating the NF-κB pathway [10]. However, it remains uncertain whether there is any relationship between HIFs and TNFα in ECs, particularly due to the fact that these two pathways share a common property to promote VEGF production and angiogenesis. To this end, we first analyzed such a potential relationship in ECs, together with VEGF and NF-κB pathways, by utilizing the publically-available databases of gene expression profiling (GEP). The datasets used includes one for differentiation of human embryonic stem cells (hESC) to mature ECs (Exp HUVEC vs ESC - James - 12 - MAS5.0 - u133p2) [28] and another for comparison between freshly-isolated ECs from vein (human umbilical vein endothelial cells, HUVECs) and artery (human umbilical artery endothelial cells, HUAECs) (Normal Endothelial Cells HUAEC/HUVEC - Luttun - 38 - MAS5.0 - u133p2) [29]. As shown in Figure 1A, the heatmap displayed that there existed numerous positive correlations (red color) in gene expression between a) *HIF1A* and *EPAS1* (HIF-2α; area #1), and b) *HIF1A* or *EPAS1* and *TNF* or its receptors (e.g., *TNFRSF1B*/TNF-R2; area #2). It is also noteworthy that although both *HIF1A* and *EPAS1* positively correlated only with *KDR* (VEGFR2) among all VEGFs and their receptors, there were a number of positive cross-links between *TNF* or its receptors (both *TNFRSF1A*/TNF-R1 and *TNFRSF1B*) and VEGFs (e.g., *VEGFA* and *VEGFC*) or their receptors (*FLT1*/VEGFR1 and particularly *KDR*; area #3). They were also closely associated with the NF-κB pathway (e.g., *RIPK1*, *NFKB2*, *RELB*, *IKBKG*/NEMO, *TNFAIP3*/A20; area #4). When a comparison was made between HUVEC and HUAECs (Figure 1B), much less positive correlations were observed in areas #2 - 4, while negative correlations (blue color) were instead clearly increased. These results raise a possibility that a network involving four essential angiogenesis-driving signaling pathways including HIF (especially α subunits), TNF, VEGF, and NF-κB might be required for development of embryonic stem cells to ECs, while it might then be largely silenced after maturation of ECs no matter where they localize (e.g., in vein or artery). Of note, only a few correlations between *ARNT* (HIF-1β) and other genes were observed in both settings (Figure 1A and 1B), probably reflecting its constitutively stable feature. Interestingly, as shown in the volcano plots, the most significantly expressed gene was the pro-inflammatory cytokine *CXCL14* in hESC vs HUVEC (Supplementary Figure S1A), while it was *KDR* (encoding VEGFR2, the major VEGF receptor) in HUVEC vs HUAEC (Supplementary Figure S1B). Moreover, the patterns of individual gene expression were also markedly different, even opposite, between these two settings i.e., hESC vs HUVEC (Supplementary Figure S2A) and HUAEC vs HUVEC (Supplementary Figure S2B). Overall, a majority of genes involved in the HIF, TNF, VEGF, and NF-κB pathways were turned off once hESCs differentiated to mature ECs, while only some genes (e.g., *HIF1A*, *TNFRSF1A*, *MAP3K14*/NIK) likely remained activated in HUVECs (assuming under lower-oxygenated conditions) when compared to HUAVCs.

To examine the differences between HIF-1α- and HIF-2α-related genes in ECs, the gene ontology (GO) analysis was then performed to classify the genes whose expression correlated with either *HIF1A* or *EPAS*1 in both arterial and venous ECs from diverse locations, including cultured ECs of aorta, coronary artery, hepatic artery and vein, iliac artery and vein, pulmonary artery and vein, umbilical artery and vein, as well as freshly isolated ECs of umbilical artery and vein (Normal Endothelial Cells HUAEC/HUVEC - Luttun - 38 - MAS5.0 - u133p2) [29]. Notably, the most significant *HIF1A*- and *EPAS1*-correlated genes fell into two functionally distinct repertoires. The *HIF1A*-related genes were more relevant to EC development and angiogenesis (Figure 1C), while the *EPAS1*-related genes were however associated more with mitochondrial energy metabolism that is known to be required for HIF activation [30], blood vessel remodeling, inflammatory responses, etc. (Figure 1D). Together, these results support a notion that although both HIF-1α and HIF-2α play functional roles in promoting angiogenesis, they might function via activation of the distinct sets of target genes.

### Hypoxia-induced angiogenesis is associated with HIF activation and VEGF production in ECs

**Figure 2:**
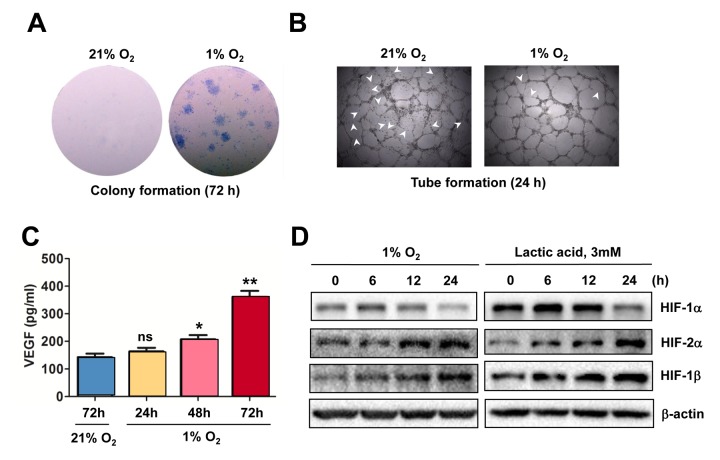
**Hypoxia induces angiogenesis of ECs, in association with VEGF production and HIF activation.** (**A**, **B**) HUVECs were cultured in ECGM medium containing 2% serum for the indicate intervals under either hypoxic (1% O_2_) or normoxic (21% O_2_) condition, after which cells were subjected to the following assays, including (**A**) colony formation assay (72 hrs) and (**B**) Matrigel-based tube formation assay (24 hrs). Representative microscopic images for at least three independent experiments were shown. Arrowheads indicate unclosed loops of vascular structure. (**C**) When HUVECs were cultured as described above under hypoxic (1% O_2_) or normoxic (21% O_2_) condition, medium was harvested at the indicated intervals and subjected to an ELISA assay to determine absolute amount of VEGF (pg/ml). Values represent the means ± SD for at least three independent experiments performed in triplicate. *P < 0.05 and **P < 0.01 for comparison with control (72 hrs under 21% O_2_); ns = not significant. (**D**) HUVECs were cultured under hypoxic (1% O_2_) condition (left panels) or exposed to the chemical hypoxia mimetic lactic acid (3 mM) for the indicated intervals (6 - 24 hrs), after which Western blot analysis was performed to monitor expression of HIF-1α, HIF-2α, and HIF-1β. Blots were reprobed for β−actin as loading control.

To confirm the causal link between HIF activation and angiogenesis in response to hypoxia, the colony formation assay was first performed to examine the effect of hypoxia on proliferation of ECs. As shown in Figure 2A, culturing HUVECs under 1% O_2_ condition led to a sharp increase in both number and size of colonies, compared to 21% O_2_ as control. Moreover, exposure of HUVECs to 1% O_2_ also enhanced their tube-forming capacity, reflected by fine vascular network with less unclosed loops (arrowhead; Figure 2B). Further, an ELISA assay was carried out to determine the amount of VEGF secreted by ECs. As shown in Figure 2C, exposure to 1% O_2_ resulted in an increase in VEGF production by HUVECs in a time-dependent manner. Moreover, Western blot analysis revealed that exposure to 1% O_2_ also resulted in a time-dependent increase in the protein levels of HIF-1α, HIF-2α, and HIF-1β (Figure 2D, left), and analogous results were obtained when lactic acid was utilized to mimic hypoxia (right). Therefore, these results indicate that hypoxia promotes angiogenesis via autocrine of VEGF by ECs, an event in association with HIF activation.

### Knockdown of either HIF-1α or HIF-2α prevents hypoxia-induced VEGF production and angiogenesis

**Figure 3:**
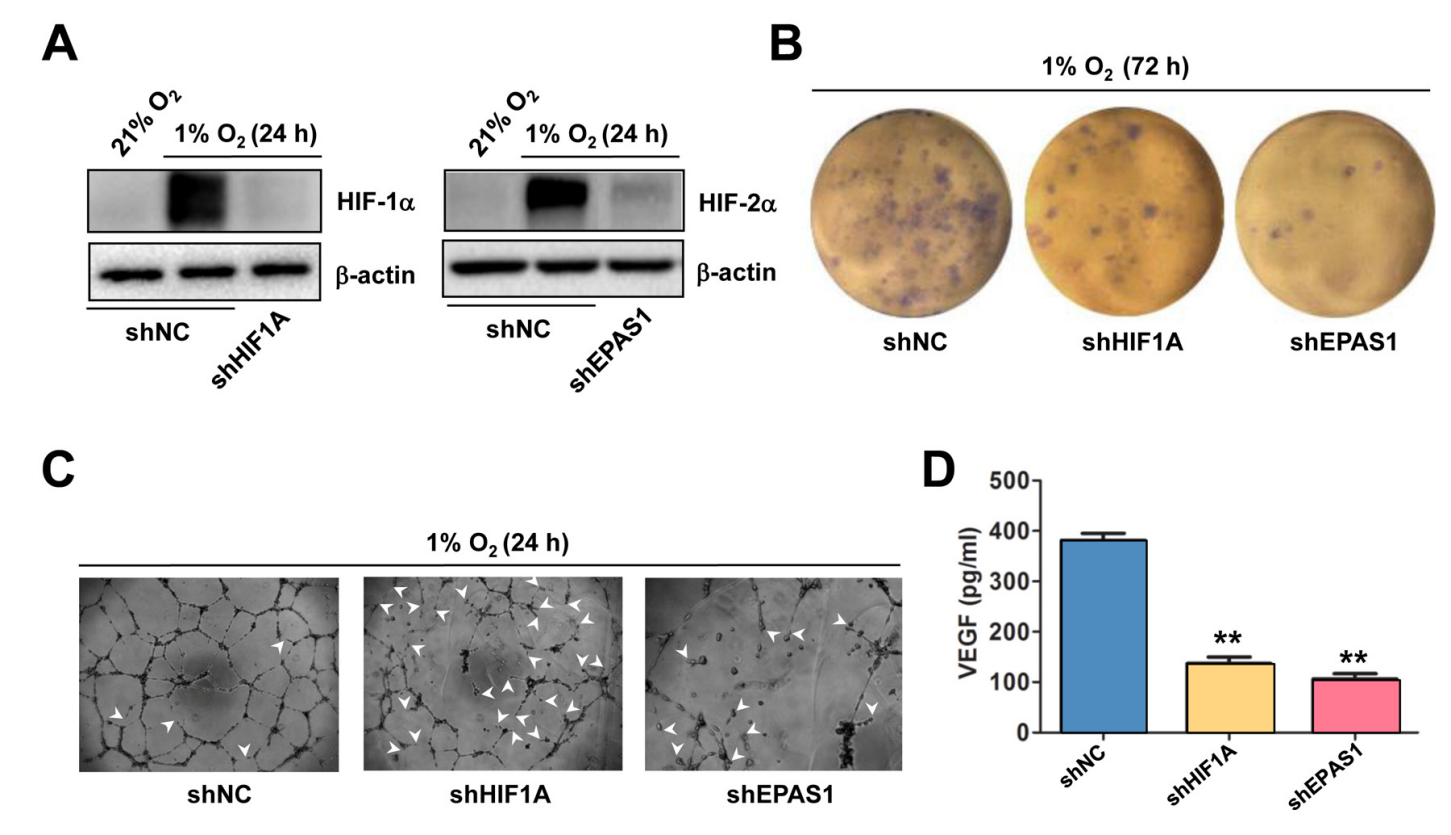
**Knockdown of either HIF-1α or HIF-2α impairs hypoxia-induced angiogenesis and VEGF production in ECs.** (**A**) HUVECs were transfected with pLKO.1 constructs encoding shRNA specifically targeting HIF-1α and HIF-2α, as well as scramble sequence as negative control (shNC). As the basal levels of HIF-1α or HIF-2α in HUVECs were relatively low, these transfected cells were exposed to 1% O_2_ (21% O_2_ as normoxia control) for 24 hrs, after which Western blot analysis was performed to confirm shRNA knockdown of HIF-1α and HIF-2α, respectively. (**B**, **C**) HUVECs expressing HIF-1α or HIF-2α shRNA were then exposed to 1% O_2_ for the indicated intervals, followed by colony formation assay (**B**, 72 hrs) and Matrigel-based tube formation assay (**C**, 24 hrs). Representative microscopic images for at least three independent experiments were shown. Arrowheads indicate unclosed loops of vascular structure. (**D**) In parallel, the VEGF level was measured by ELISA assay after cultured for 72 hrs under 1% O_2_. Values represent the means ± SD for at least three independent experiments performed in triplicate. **P < 0.01 for comparison with shNC control.

The functional role of HIF-1α and HIF-2α in hypoxia-induced angiogenesis was then examined. To this end, HIF-1α and HIF-2α were knocked down in HUVECs, using shRNA specifically targeting *HIF1A* and *EPAS1*, respectively. Western blot analysis confirmed knockdown of *HIF1A* and *EPAS1* dramatically prevented robust expression of HIF-1α (Figure 3A, left) and HIF-2α (right) in HUVECs exposed to 1% O_2_. Using these cells, the colony and tube formation assays were performed to evaluate the functional role of HIF-1α and HIF-2α in ECs. Indeed, prevention of either *HIF1A* or *EPAS1* expression sharply suppressed growth of HUVECs under 1% O_2_ condition, while knocking down *EPAS1* was even more effective than knockdown of *HIF1A* (Figure 3B). Consistently, whereas knocking down either of them markedly impaired the capacity of HUVECs to form vascular network under 1% O_2_ condition, manifested by both reduced number of tubes and increased unclosed loops (arrowhead; Figure 3C). Similar results were observed in HUVECs with shRNA knockdown of *ANRT* (Supplementary Figure S3A). Last, knockdown of *HIF1A* or *EPAS1* also significantly attenuated production of VEGF by HUVECs (Figure 3D). Together, these findings argue that hypoxia induces angiogenesis through inducing autocrine of VEGF by ECs via activation of the HIF pathway.

### LDL inhibits activation of both HIF and NF-κB pathways in ECs exposed to hypoxia

**Figure 4:**
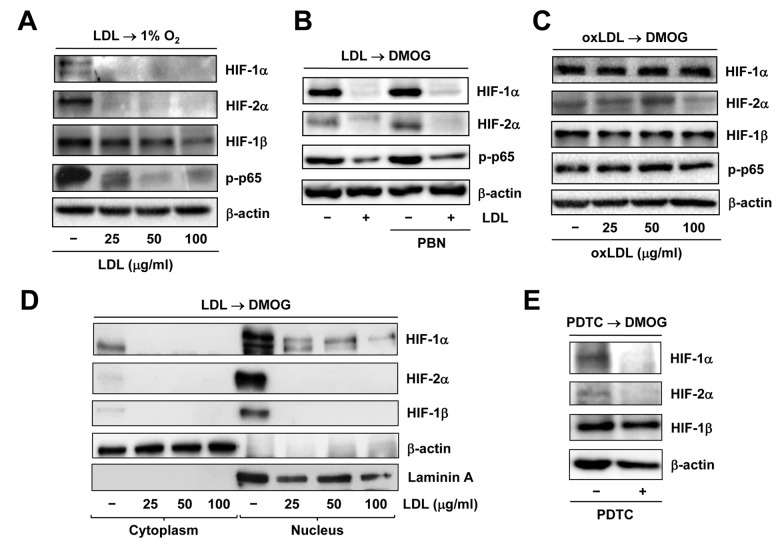
**Native LDL, but not oxLDL, inhibits activation of HIF and NF−κB signals induced by hypoxia.** HUVECs were treated as follows: (**A**) pre-treated with LDL (100 μg/ml) for 48 hrs, followed by incubation under hypoxic (1% O_2_) condition for additional 48 hrs; (**B**) pre-treated with LDL (100 μg/ml) in the presence or absence of the free radical scavenger PBN (2 mM) for 48 hrs, and then exposed to the PHD inhibitor DMOG (1 μM) for additional 72 hrs; (**C**) pre-treated with the indicated concentrations of oxidized LDL (oxLDL, μg/ml) for 48 hrs, followed by DMOG (1 μM) for additional 72 hrs. After treatment, Western blot analysis was performed to monitor expression of HIF-1α, HIF-2α, and HIF-1β, as well as phosphorylation of NF-κB p65 (S536). (**D**) Alternatively, HUVECs were treated as described in panel 4B, after which cytoplasmic and nuclear fractions were separated and subjected to Western blot analysis for monitoring nuclear translocation of HIF-1α, HIF-2α, and HIF-1β. Blots were reprobed for β-actin and laminin A as loading controls for cytoplasmic and nuclear fractions, respectively. (**E**) HUVECs were pre-incubated with the NF-κB inhibitor PDTC (100 μM) for 4 hrs, followed by DMOG (1 μM) for additional 72 hrs, after which the protein levels of HIFs were monitored by Western blot analysis.

We have previously demonstrated that LDL impairs angiogenesis by disrupting several pro-angiogenic signals such as SDF/CXCR4 [31] and VEGF/VEGFR2 [27]. Recently, we have found that LDL also acts to down-regulate HIF-1β expression by inactivating NF-κB, followed by down-regulation of HIF-1α and HIF-2α, which in turn contributes to the anti-angiogenic effect of LDL [18]. Consistent with these findings, Western blot analysis revealed that pre-treatment with native LDL sharply diminished expression of HIF-1α, HIF-2α, and to a lesser extent HIF-1β, as well as phosphorylation of p65, reflecting NF-κB inactivation [32], in HUVECs cultured under 1% O_2_ condition (Figure 4A). Similarly, LDL pre-treatment largely decreased accumulation of HIF-1α and HIF-2α in HUVECs treated with the PHD inhibitor DMOG [33], in association with reduced p65 phosphorylation (Figure 4B). However, oxidized LDL (oxLDL) failed to do so in HUVECs exposed to DMOG (Figure 4C). Neither LDL nor oxLDL were able to down-regulate HIF-1β under 21% O_2_ condition (Supplementary Figure S3B and 3C). Moreover, the free radical scavenger PBN [34] could not rescue these events mediated by LDL (Figure 4B), supporting a notion that oxidation might be not required for LDL to inhibit activation of the HIF and NF-κB pathways. Whereas treatment with DMOG resulted in an increase in the protein levels of HIF-1α, HIF-2α, and HIF-1β in nucleus where they form the active heterodimer complexes to trigger transcription of target genes, this event was sharply blocked by pre-treatment with LDL (Figure 4D and Supplementary Figure S3D). Similarly, pre-incubation with the NF-κB inhibitor PDTC [35] also markedly reduced the protein levels of HIF-1α and HIF-2α in ECs treated with DMOG, but only modestly affected HIF-1β protein level (Figure 4E). Together, these results argue that LDL, rather than oxLDL, is capable to block hypoxia-induced activation of the HIF pathway in ECs, in association with NF-κB inactivation.

### TNFα exposure results in a distinct GEP for relationship among the TNF, HIF, NF-κB and VEGF pathways in ECs

**Figure 5:**
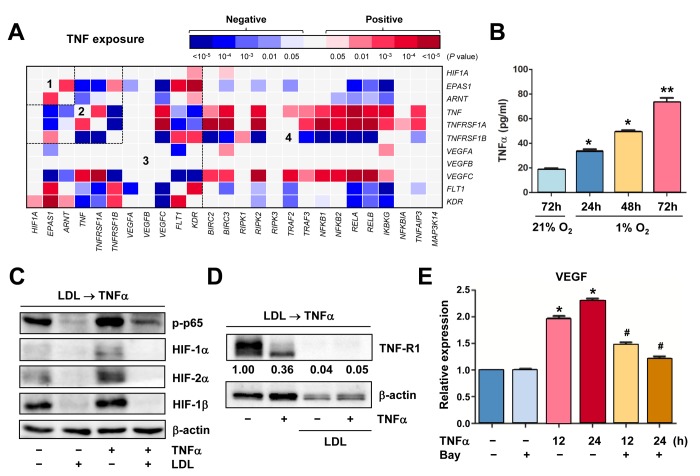
**Hypoxia induces autocrine of TNFα in ECs, which in turn activates HIFs and NF-κB to produce VEGF.** (**A**) Using the same approach as described in Figure 1A and 1B, the heatmap was generated by analyzing a dataset involving gene expression profiling (GEP) in HUVECs stimulated by TNFα (dataset, Exp HUVEC TNF-alpha - Kodama - 25 - MAS5.0 - u133p2). Numbers in the heatmaps indicate the areas (outlined by dash line) clustered for each pathway. (**B**) HUVECs were cultured under hypoxic (1% O_2_) or normoxic (21% O_2_) condition as described in Figure 2C, an ELISA assay was performed to determine absolute amount of TNFα (pg/ml) in medium harvested at the indicated intervals. Values represent the means ± SD for at least three independent experiments performed in triplicate. *P < 0.05 and **P < 0.01 for comparison with control (72 hrs under 21% O_2_). (**C**, **D**) HUVECs were pre-treated with LDL (100 μg/ml) for 48 hrs, followed by TNFα (50 ng/ml) for additional intervals as below, after which Western blot analysis was performed to monitor S536 phosphorylation of NF-κB p65 (5 min) and expression of HIFs (**C**, 4 hrs) and TNF-R1 (**D**, 4 hrs). Blots for TNF-R1 were densitometrically quantified by using ImageJ software. Values indicate fold change after normalization to β-actin. (**E**) After pre-treated with the IKK inhibitor Bay 11-7082 (10 μM) for 4 hrs, HUVECs were exposed to TNFα (50 ng/ml) for additional 24 hrs. Real-time PCR analysis was then performed to determine expression of VEGF in ECs the indicated intervals. Values represent the means ± SD for at least three independent experiments performed in triplicate. *P < 0.05 for comparison with untreated control; ^#^*P* < 0.05 for comparison with TNFα alone at the same time point.

It is known that TNFα activates HIFs in macrophages. However, the role of TNFα in ECs remains uncertain, particularly regarding angiogenesis. To this end, the GEP database of ECs exposed to TNFα [36] was analyzed, in order to seek for potential relationship among TNF, HIF, NF-κB, and VEGF. As expected, TNFα treatment rapidly induced NF-κB activation, reflected by robust expression of *NFKBIA* (encoding IκBα) and *TNFAIP3* (Supplementary Figure S4A), two direct downstream targets of NF-κB. As in shown in Figure 5A, exposure of ECs to TNFα resulted in a distinct heatmap for gene expression correlations involving these pathways, which was clearly different from that for EC development (Figure 1A) or for ECs from artery vs vein (Figure 1B). Of note, *EPAS1*, rather than *HIF1A*, was co-expressed with *ARNT* (area #1). Positive correlations were also observed between *HIF1A* or *ARNT* and *KDR*, as well as *EPAS1* and VEGF receptors (both *FLT1* and *KDR*; area #3). Moreover, expression of VEGFs (particularly *VEGFC*) correlated with *TNF* and its receptor *TNFRSF1A*, in association with activation of the NF-κB pathways (area #4). The positive correlations were also observed between *HIF1A* and *KDR* or *EPAS1* and *FLT1* (Supplementary Figure S4B), as well as *HIF1A* and the glycolytic activator *PFKFB3* (Supplementary Figure S4C) that is known to promote angiogenesis [37]. In contrast, *HIF1A* or *EPAS1* adversely correlated with *NOS3*, which is involved in synthesis of the angiogenic mediator NO [38], and OTUD7B, a negative regulator of the non-canonical NF-κB pathway [16]. However, exposure of ECs to VEGF yielded much less correlations among these pathways (Supplementary Figure S5A), when compared to TNFα treatment (Figure 5A). Interestingly, positive correlations between *HIF1A* (but neither *EPAS1* nor *ARNT*) and VEGF receptors (both *FLT1* and *KDR*) were observed in ECs exposed to VEGF (Supplementary Figure S5B), while all three subunits (particularly *EPAS1*) positively correlated with VEGF receptors in ECs treated with TNFα (Figure 5A). Together, these results suggest that TNFα activates ECs likely via a distinct signaling network that links itself and its receptors with HIFs and NF-κB, as well as VEGFs and particularly their receptors, different from those for EC differentiation, ECs located at artery vs vein, or stimulated with other pro-angiogenic factors (e.g., VEGF).

### Hypoxia induces TNFα production by ECs that in turn activates the HIF pathway, while this autocrine loop is disrupted by LDL via down-regulation of TNF-R1

Because the GEP analyses showed that TNF was up-regulated in ECs under certain circumstances (e.g., Figures 1A and 5A), we thus examine whether hypoxia would induce production of TNFα by ECs. Indeed, an ELISA assay revealed that hypoxia induced a marked increase in production of TNFα by HUVECs in a time-dependent manner (Figure 5B). In turn, treatment with TNFα sharply induced NF-κB activation (e.g., p65 phosphorylation) and HIF-1β expression, in association with up-regulation of HIF-2α and to a lesser extent HIF-1α (Figure 5C, lane 3 vs 1). These results suggest an autocrine loop of TNFα in ECs under hypoxic conditions. However, pre-treatment with LDL almost completely eliminated both basal and TNFα-induced NF-κB activation and expression of all three HIF subunits (Figure 5C, lane 2 vs 1 and 4 vs 3, respectively). Moreover, although LDL failed to reduce, rather increased, TNFα production by ECs (Supplementary Figure S5C), it sharply down-regulated TNF-R1 that preferentially accounts for TNFα-induced NF-κB activation [39], in the presence or absence of TNFα (Figure 5D). Interestingly, it was noted that exposure of ECs to TNFα also clearly reduced TNF-R1, presumably reflecting a negative feedback response to TNFα stimulation via an unknown mechanism. Last, exposure to TNFα induced expression of VEGF in HUVECs, an event significantly blocked by the NF-κB inhibitor Bay 11-7082 [40] (Figure 5E). Together, these findings argue that hypoxia might induce angiogenesis via an autocrine loop of TNFα in ECs, which involves VEGF production via activation of the HIF and NF-κB pathways. They also raise a possibility that LDL impairs hypoxia-induced angiogenesis by interfering with this signaling network in ECs, primarily due to down-regulation of TNF receptors (e.g., TNF-R1), rather than TNFα.

### LDL down-regulates multiple key components of both canonical and non-canonical NF-κB pathways

**Figure 6:**
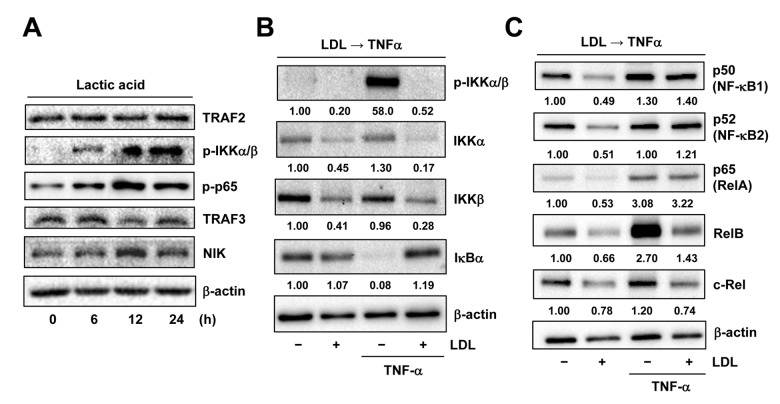
**LDL inactivates TNFα-induced NF-κB activation via down-regulation of its key signaling components.** (**A**) HUVECs were treated with 3 mM lactic acid as described in Figure 2D, after which Western blot analysis was performed to assess activation of the canonical (e.g., TRAF2 expression as well as IKKα/β and p65 phosphorylation) and non-canonical (e.g., expression of TRAF3 and NIK) NF-κB pathways. (**B**, **C**) HUVECs were pre-treated with LDL (100 μg/ml) for 48 hrs, followed by TNFα (50 ng/ml) for additional intervals as below, after which Western blot analysis was carried out to monitor phosphorylation of IKKα/β (Ser176/180, 5 min) as well as expression of multiple key components of both canonical and non-canonical NF-κB pathways, including IKKα, IKKβ, IκΒα (5 min), p50 (NF-κB1), p52 (NF-κB2), p65 (RelA), RelB, and c-Rel (4 hrs). All blots were densitometrically quantified, and values indicate fold increase after normalization to β-actin.

As LDL was able to disrupt the autocrine loop of TNFα likely via down-regulation of TNF-R1, a question then arose how LDL would act to down-regulate TNF-R1. As described above, expression of *TNFRSF1A* (encoding TNF-R1) significantly correlated with activation of NF-κB, rather than HIFs, in ECs exposed to TNFα (Figure 5A). In this context, the effect of LDL on expression of the key components involving the canonical and non-canonical NF-κB pathways was then examined. Similar to that we previously observed in ECs exposed to 1% O_2_ [18], the chemical mimetic lactic acid could also activate the canonical NF-κB pathway in HUVECs, reflected by increased phosphorylation of IKKα/β and p65 [32], without affecting TRAF2 protein level (Figure 6A, upper panels). However, exposure to the PHD inhibitors (e.g., DMOG, CoCl_2_), which mimic hypoxia by causing accumulation of HIF-1α or HIF-2α due to prevention of their hydroxylation and proteasomal degradation [2], failed to induce NF-κB activation (data not shown). These results suggest that lactic acid might represent an alternative approach to mimic hypoxia more appropriately, in order to evaluate the cross-talk between HIF and NF-κB signaling pathways. Whereas TRAF3 is known to bind to NIK and lead to its ubiquitination and proteasomal degradation [32], we observed that lactic acid also reduced TRAF3 but increased NIK, reflecting activation of the non-canonical NF-κB pathway in HUVECs (Figure 6A, lower panels). Similarly, exposure to TNFα also resulted in a sharp increase in phosphorylation of IKKα/β (Figure 6B) as well as up-regulated RelB (Figure 6C) that binds to NF-κB2/p52 to activate the non-canonical NF-κB pathway [19]. Of note, pretreatment with LDL down-regulated the basal levels of multiple key components involving these two NF-κB pathways, including IKKα/IKKβ, p50, p52, p65 (RelA), RelB and c-Rel. LDL pretreatment also blocked TNFα-induced IKKα/β phosphorylation and RelB up-regulation, as well as down-regulated IKKα/IKKβ, RelB and c-Rel in HUVECs exposed to TNFα, while reversed down-regulation of IκBα by TNFα. Interestingly, LDL barely reduced the protein levels of p50, p52 and p65 in the presence of TNFα (Figures 6B and 6C). Together, these results suggest that in addition to down-regulation of TNF-R1, LDL might also disrupt the autocrine loop of TNFα in ECs by inactivating both canonical and non-canonical NF-κB pathways via down-regulation of their key components.

### LDL-mediated impairment of hypoxia-induced angiogenesis involves multiple HIF-dependent downstream targets

**Figure 7:**
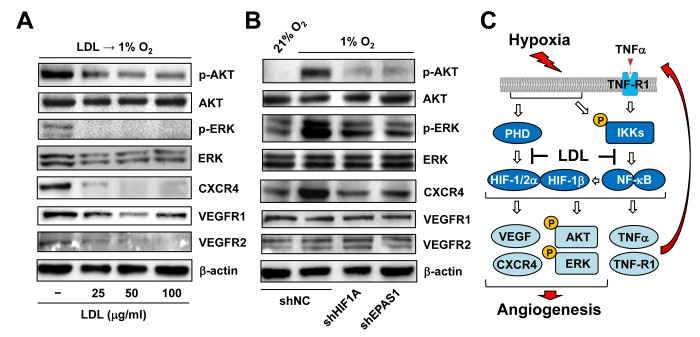
**LDL-mediated impairment of hypoxia-induced angiogenesis involves a broad spectrum of HIF-dependent signaling pathways.** (**A**) HUVECs were exposed to indicated concentrations of LDL (25 - 100 μg/ml) for 48 hrs, and then cultured under hypoxic (1% O_2_) condition for additional 48 hrs. (**B**) HUVECs with shRNA knockdown of HIF-1α or HIF-2α were exposed to 1% O_2_ (21% O_2_ as normoxia control) for 24 hrs. After treatment, Western blot analysis was performed to monitor phosphorylation of AKT (S473), ERK1/2 (T202/Y204), CXCR4, VEGFR1, and VEGFR2. Blots were reprobed for β-actin as loading control. (**C**) A schematic diagram for the mechanism by which hypoxia induces angiogenesis via an autocrine loop of TNFα and resulting activation of the self-regulatory TNFα/NF-κB/HIF/VEGF signaling network in ECs, as well as the potential mechanism of action for anti-angiogenic property of LDL by which a) LDL might impair the autocrine loop of TNFα via down-regulation of its receptor TNF-R1, rather than TNFα itself; and b) LDL disrupts the TNFα-NF-κB-HIF-VEGF signaling cascade via down-regulation of multiple key components of both canonical and non-canonical NF-κB pathways.

Last, we sought to find out potential downstream events of HIFs responsible for anti-angiogenic activity of LDL. To this end, the effect of LDL on major targets and pathways that are known to be involved in angiogenesis was examined in ECs exposed to hypoxia. As shown in Figure 7A, pre-treatment with LDL sharply reduces phosphorylation (activation) of AKT and ERK1/2, as well as expression of VEGF receptors (e.g., VEGFR1 and VEGFR2) and CXCR4 in HUVECs cultured under 1% O_2_. HUVECs with knockdown of *HIF1A* or *EPAS1* were then utilized to determine whether disruption of the HIF pathway would reproduce these effects of LDL. Indeed, prevention of either HIF-1α or HIF-2α expression (Figure 3A) also markedly diminished activation of both AKT and ERK1/2, as well as CXCR4 expression induced by hypoxia (Figure 7B, upper panels). However, they failed to affect the protein levels of VEGFR1 and VEGFR2 (Figure 7B, lower panels). Together, these findings argue that the mechanism by which LDL impairs hypoxia-induced angiogenesis via inactivation of the TNFα/NF-κB/HIF/VEGF signaling network might involve a broad spectrum of HIF-dependent downstream events in ECs.

## DISCUSSION

The transcription factor HIFs are central regulators of adaptive responses to hypoxia in various physiological and pathological processes, including angiogenesis and inflammation [41]. In the former, HIFs act as a master switch during angiogenesis through inducing production of VEGFs and other growth factors by diverse types of cells (particularly ECs and macrophages) [42], to recover blood supply and restore oxygenation in hypoxic tissues (e.g., caused by ischemic diseases, such as stroke and myocardial infarction) [43,44]. However, such a protective role is often impaired due to hyperlipidemia that is common in patients with aging-related disorders, such as atherosclerotic diseases [24–27]. In the latter, HIFs (HIF-1α in particular) plays an important role in inflammation through promoting production of TNFα and other cytokines or chemokines by immune cells (especially macrophages) [45]. As TNFα also serves as a potent pro-angiogenic factor, HIF-1α-mediated production of TNFα by macrophages could be considered as an alternative mechanism for increased angiogenesis in certain circumstances [46], such as within atherosclerotic plaques where macrophages are the most abundant cellular component [47]. However, the role of HIFs and NF-κB (another major transcription factor related to both TNFα and hypoxia) in the potential cross-link between pro-inflammatory TNFα and pro-angiogenic VEGFs remains to be defined in ECs whose activation and dysfunction are the hallmark of age-related vascular diseases (e.g., atherosclerosis [48] and neovascular macular degeneration [49]). Here we showed for the first time that hypoxia could induce autocrine of TNFα by ECs, which in turn activated the HIF pathways via a NF-κB-dependent process, thereby promoting VEGF production by ECs and angiogenesis, a process that might not need macrophages. Notably, the anti-angiogenic property of LDL likely involved disruption of this self-regulatory HIF-centered signaling network in ECs. Thus, these findings might provide a new insight into the mechanism underlying hypoxia-induced angiogenesis as well as its defect in patients with hyperlipidemia-related diseases.

Whereas immune cells (particularly macrophages) are considered as the major source of TNFα [45], very few studies has reported that ECs could also secret TNFα, which forms an autocrine loop for regulation of EC functions e.g., production of NO and ICAM in inflammation [50,51]. Unexpectedly, we observed that exposure to hypoxia was able to induce marked production of TNFα by ECs, consistent with its mRNA expression in ECs as well as its up-regulation in ECs treated with TNFα or VEGF as revealed by the GEP analyses (not shown). Moreover, it was also noted that expression of both TNF and its receptor TNF-R1 (encoded by *TNFRSF1A*) positively correlated with activation of the NF-κB pathways, rather than HIFs, in ECs treated with TNFα. These observations suggest that under hypoxia, the autocrine loop of TNF production by ECs might depend upon activation of NF-κB, but not HIFs. Like hypoxia, TNFα also activated both NF-κB (including canonical and non-canonical) and HIF pathways. As consequence, these events led to a marked increase in HIF-dependent production of angiogenic factors (e.g., VEGF [52,53] and CXCR4 [31]) by ECs, in association with activation of other pro-angiogenic pathways (e.g., ERK1/2 and AKT [54]). In conjunction with our pervious observation that hypoxia could induce up-regulation of all three HIF subunits via an NF-κB-dependent process [18], these findings argue strongly that hypoxia acts to initiate and/or promote angiogenesis via a self-regulatory TNFα/NF-κB/HIF/VEGF signaling network in ECs, as summarized in Figure 7C.

Although HIF-1α and HIF-2α share high similarities in their structures and amino acid compositions [2], there are numerous differences between these two HIF isoforms. For example, they are encoded by entirely different genes (i.e., *HIF1A* and *EPAS1*, respectively) [3]. In contrast to ubiquitous expression of HIF-1α in most mammalian cell types, expression of HIF-2α is highly restricted in certain cell and tissue types, especially ECs and the tissues enriched for blood vessels [12]. Although both of them can be induced by hypoxia, higher degree and longer duration of hypoxia may be required to induce HIF-2α than HIF-1α [55,56]. Moreover, the levels of both proteins are primarily controlled by PHD-catalyzed hydroxylation at proline residues (though at different sites), leading to rapid degradation via the von Hippel-Lindau (pVHL)-mediated UPS pathway [15]. However, it has recently found that HIF-2α might also be degraded via an alternative process (e.g., pVHL-independent degradation through interaction with eIF3e/INT6 under normoxia) [57]. Due to its EC-selective expression pattern, HIF-2α is considered to regulate a spectrum of genes more specific for ECs and thereby play a functional role distinct from HIF-1α [58]. Indeed, the GEP analysis revealed that although expression of *HIF1A* and *EPAS1*, but not *ARNT* (encoding HIF-1β), was correlated during EC development as well as in ECs from different locations (e.g., vein vs artery), their related genes however fell into functionally different categories in ECs. Moreover, *EPAS1*, but not *HIF1A*, became correlated with *ARNT* in ECs exposed to TNFα, raising a possibility that HIF-2α might play a predominant role in this setting. Consistent with this finding, we observed that knockdown of *EPAS1* in ECs was more potent than *HIF1A* knockdown in inhibition of hypoxia-induced angiogenesis (e.g., cell proliferation, tube formation, and to a lesser extent VEGF production). However, further studies specifically designed to distinguish the roles of HIF-2α from HIF-1α in angiogenesis (especially induced by hypoxia) are required for addressing this issue.

Several groups including ours have documented that LDL impairs angiogenesis [24–27]. In previous study, we have demonstrated a potential mechanism underlying anti-angiogenic property of LDL, by which LDL acts to suppress HIF-1β expression at transcriptional level via NF-κB inactivation, thereby resulting in down-regulation of HIF-1α and HIF-2α in ECs [18]. The present study might gain further insights into this mechanism by providing new evidence supporting an autocrine loop of TNFα in ECs under hypoxia. We also found that pre-treatment with LDL almost completely shut down the HIF and NF-κB pathways under basal untreated condition as well as after TNFα treatment, resulting in a marked reduction in VEGF production by ECs and inactivation of a broad spectrum of HIF-dependent downstream signals (e.g., ERK1/2, AKT, and CXCR4) [31,54]. However, while LDL could also down-regulate VEGFR1 and VEGFR2 expression, knockdown of either HIF-1α or HIF-2α failed to affect their protein levels. These observations raise a possibility that other HIF-independent events (e.g., down-regulation of VEGF receptors) might also contribute to the anti-angiogenic property of LDL. Of note, disruption of the TNFα autocrine loop by LDL was most likely stemmed from down-regulation of the TNFα receptor TNF-R1 that is primarily responsible for NF-κB activation [39], rather than blockade of TNFα production by ECs. In addition, we also observed that LDL prevented TNFα-triggered activation of both canonical and non-canonical NF-κB pathways via down-regulation of their key signaling components. Together, the mechanisms underlying the anti-angiogenic property of LDL are summarized in Figure 7C.

In conclusion, we observed for the first time that hypoxia could induce autocrine of TNFα by ECs, at least in the *in vitro* model of angiogenesis used in the present study. We also provide evidence supporting a notion that hypoxia facilitates angiogenesis via activation of a self-regulatory HIF-centered signaling network involving interactions among four major pathways in ECs, including TNFα, NF-κB, HIFs, and VEGFs. Finally, we also provide new insights into the mechanism for anti-angiogenic property of LDL, in which LDL might act to turn off the TNFα/NF-κB/HIF/VEGF signaling cascade in ECs by down-regulation of TNF-R1 as well as multiple key components of the NF-κB pathways, leading to inactivation of a broad spectrum of HIF-dependent downstream events. However, whereas low percentage of O_2_ (e.g., 0.5 - 2%, with 21% O_2_ in the atmosphere used as control) has been widely used in the vast majority of *in vitro* studies involving hypoxia, there is a caveat that these concentrations of O_2_ in the air might not necessarily reflect the concentrations of O_2_ dissolved in the media or in the body where O_2_ concentrations usually range from 1 to 12%. Therefore, these *in vitro* observations in the present study warranted further validation in succeeding investigation *in vivo*. Nevertheless, the findings of the present study likely provide new evidence supporting a potential link between angiogenesis and inflammation in ECs, two events essential for aging-related diseases (e.g., atherosclerosis ) [59] in which hyperlipidemia represents a common and major risk factor.

## MATERIALS AND METHODS

### Cell culture

Human umbilical vein endothelial cell (HUVEC) Pooled Pellet (1x10^6^ cells in RNAlater, Cat # C-14011), which contains ECs isolated from more than two donors, was obtained from PromoCell (Heidelberg, Germany). Each experiment was performed in at least three different batches of pooled HUVECs. As described previously [54], cells were cultured for up to 6 passages in endothelial cell growth medium (ECGM, PromoCell) containing 2% serum, 0.1 ng/ml human epidermal growth factor (EGF), 1 ng/ml basic fibroblast growth factor (bFGF), 90 μg/ml heparin and 1 μg/ml hydrocortisone. Cells were maintained at 37°C in a humidified 5% CO_2_ incubator at 21% O_2_. 1% O_2_ condition used for virtually all hypoxic experiments was achieved in a chamber with continuous infusion of pre-tested gas mixture containing 95% N_2_ and 5% CO_2_. Alternatively, lactic acid, cobalt, and DMOG (Sigma-Aldrich, St. Louis, MO) were used as chemical hypoxia mimetics.

### Reagents

LDL and recombinant human tumor necrosis factor-α (TNFα) were purchased from Invitrogen (Carlsbad, CA) and Peprotech (Rocky Jill, NJ), respectively. The free radical scavenger phenyl-N-tert-butyInitrone (PBN), the NF-κB inhibitor pyrrolidine dithiocarbamic acid (PDTC), the IκB kinase (IKK) inhibitor Bay 11-7082, the prolyl-hydroxylase inhibitor dimethyloxalylglycine (DMOG), and lactic acid were obtained from Sigma-Aldrich. The antibodies used in this study include mouse anti-human HIF-1α monoclonal antibody (BD Transduction Laboratories, San Jose, CA); rabbit anti-human HIF-2α polyclonal antibody and rabbit anti-total VEGFR1 (ab32152) monoclonal antibody (Abcam, Cambridge, MA); mouse anti-human CXCR4 (Fusin) monoclonal antibody, rabbit anti-human HIF-1β/ARNT polyclonal antibody, rabbit anti-human NF-κB p65 monoclonal antibody, rabbit anti-total Akt monoclonal antibody, rabbit anti-phospho-Akt (Ser473) polyclonal antibody, rabbit anti-total extracellular signal-regulated kinase (ERK)1/2 monoclonal antibody, rabbit anti-phospho-ERK1/2 (Thr202/Tyr204) monoclonal antibody, rabbit anti-human TNFR1 monoclonal antibody, rabbit anti-human IKKα polyclonal antibody, rabbit anti-human IKKβ monoclonal antibody, rabbit anti-human phospho-IKKα/β monoclonal antibody, rabbit anti-human IκBα polyclonal antibody, rabbit anti-human p50 monoclonal antibody, rabbit anti-human p52 monoclonal antibody, rabbit anti-human p65 monoclonal antibody, rabbit anti-human c-Rel monoclonal antibody, rabbit anti-human RelB monoclonal antibody, rabbit anti-human β-actin antibody (Cell Signaling Technology, Danver, MA); human VEGF and TNFα Quantikine ELISA Kit (R&D Systems, Minneapolis, MN).

### Colony formation assay

200 HUVECs cells were seeded into individual well in 24-well plates and grew for 7 days under either 21% or 1% O_2_ condition. After washing with PBS for 3 times, cells were fixed using methanol and stained with Giemsa. The numbers of colonies were counted and photographs were captured.

### Cell proliferation and migration assays

2×10^4^ HUVECs were suspended in ECGM containing 0.5% serum and seeded into 24-well plates [54]. After incubation for 24 hours, cell viability was analyzed using the Cell Counting Kit-8 (CCK-8; Dojindo Laboratories, Kumamoto, Japan). Alternatively, cell migration assay was conducted in a modified Boyden chamber, with 24-well plates in which transwell inserts (polycarbonate membrane insert with 6.5-mm diameter and 8.0 μm pores; Corning) were placed. 2×10^4^ HUVECs were seeded into the upper compartment and incubated for 24 hours. Non-migrated cells on the top side of the surface were removed, while migrated cells on the bottom side of the membrane were counted in 12 fields.

### Matrigel-based tube formation assay

2×10^4^ HUVECs were suspended with ECGM and then seeded into Matrigel-coated wells of a 96-well plate [60]. After incubated for 24 hours, photographs were taken at low magnification (5×) with a DFC-290 camera (Leica Microsystems, Wetzlar, Germany). Unclosed tubes between two branching points were counted.

### LDL oxidation

Briefly, native LDL was dialysed against 1× PBS for 24 hours and then incubated with 10 µM CuSO_4_ for 24 hours at 37°C, followed by dialysis against 1× PBS containing 0.1 mM EDTA for 48 hours. Oxidized LDL was harvested through a 0.22 µm filter and used within one week [18].

### Plasmids and lentivirus transduction

E. coli XL1-blue (Stratagene, Heidelberg, Germany) was used for plasmid preparation, while E. coli Stbl3 (Invitrogen) were used for preparation of pLKO.1 (Addgene, Cambridge, MA), pLKO.1-shRNA-*HIF1A*, pLKO.1-shRNA-*EPAS1*, pLKO.1-shRNA-*ARNT* (Sigma-Aldrich). Recombinant lentivirus was produced as described previously [61]. HUVECs were infected with lentivirus and then sorted by flow cytometry. Western blot analysis was performed to confirm silencing of target genes.

### Western blot analysis

Whole cell lysates were prepared in NP40 lysis buffer containing protease inhibitor cocktail (Roche, Deutschland, Germany). Whole cell lysates or nuclear/cellular fractions were resolved on sodium dodecyl sulfate-polyacrylamide gels (SDS-PAGE) and blotted onto polyvinylidene fluoride (PVDF) membranes. Membranes were then blocked with 5% skimmed milk in TBS-T (tris-buffered saline containing 0.5% Tween-20, pH 7.2), followed by incubation with the primary antibodies (see “Reagents”). Blots were visualized using the Enhanced Chemiluminescence (ECL) Kit (Pierce, Life Technologies). Blots were re-probed with anti-β-actin (Cell Signaling Technology) to ensure equal loading.

### Qualitative real-time-PCR (qPCR)

qPCR analysis using the ABI PRISM 7500 Real Time PCR System (Applied Biosystems, Foster City, CA) was performed to quantify mRNA levels of target genes. Briefly, total RNA was extracted using the RNeasy Midi Kit (Qiagen, Valencia, CA) as per the manufacturer’s instructions. cDNA was synthesized from 1 µg of total RNA. Gene expression was then analyzed by two-step real-time PCR: 95^o^C for 30 seconds, followed by 40 cycles of 95^o^C for 5 seconds, and 60^o^C for 34 seconds. The following primer for *VEGF* was used: forward, 5- AGGGCAGAATCATCACGAAGT-3; backward 3-ACGGTAGGTTAGCTCTGGGA-5. The human housekeeping gene GAPDH was used as reference. All PCR reactions were performed in triplicate, and gene expression relative to *GAPDH* was calculated using the 2-ΔΔCT method.

### Statistical analysis

All statistical analyses were carried out using the GraphPad Prism 5. Values represent the means ± SD for at least three independent experiments performed in triplicate. Significance of differences between experimental variables was determined using the Student’s t test (two-sided) or One-way ANOVA with Tukey Post-Hoc Test. *P* < 0.05 was considered statistically significant.

## Supplementary Material

Supplementary Figure 1

Supplementary Figure 2

Supplementary Figure 3

Supplementary Figure 4

Supplementary Figure 5
