# Supplementary figures and images for: Impairment of hypoxia-induced angiogenesis by LDL involves a HIF-centered signaling network linking inflammatory TNFα and angiogenic VEGF
Source: Aging (Albany NY). 2019 Jan 18;11(2):328–49. doi: 10.18632/aging.101726 (PMC6366960; doi:10.18632/aging.101726)

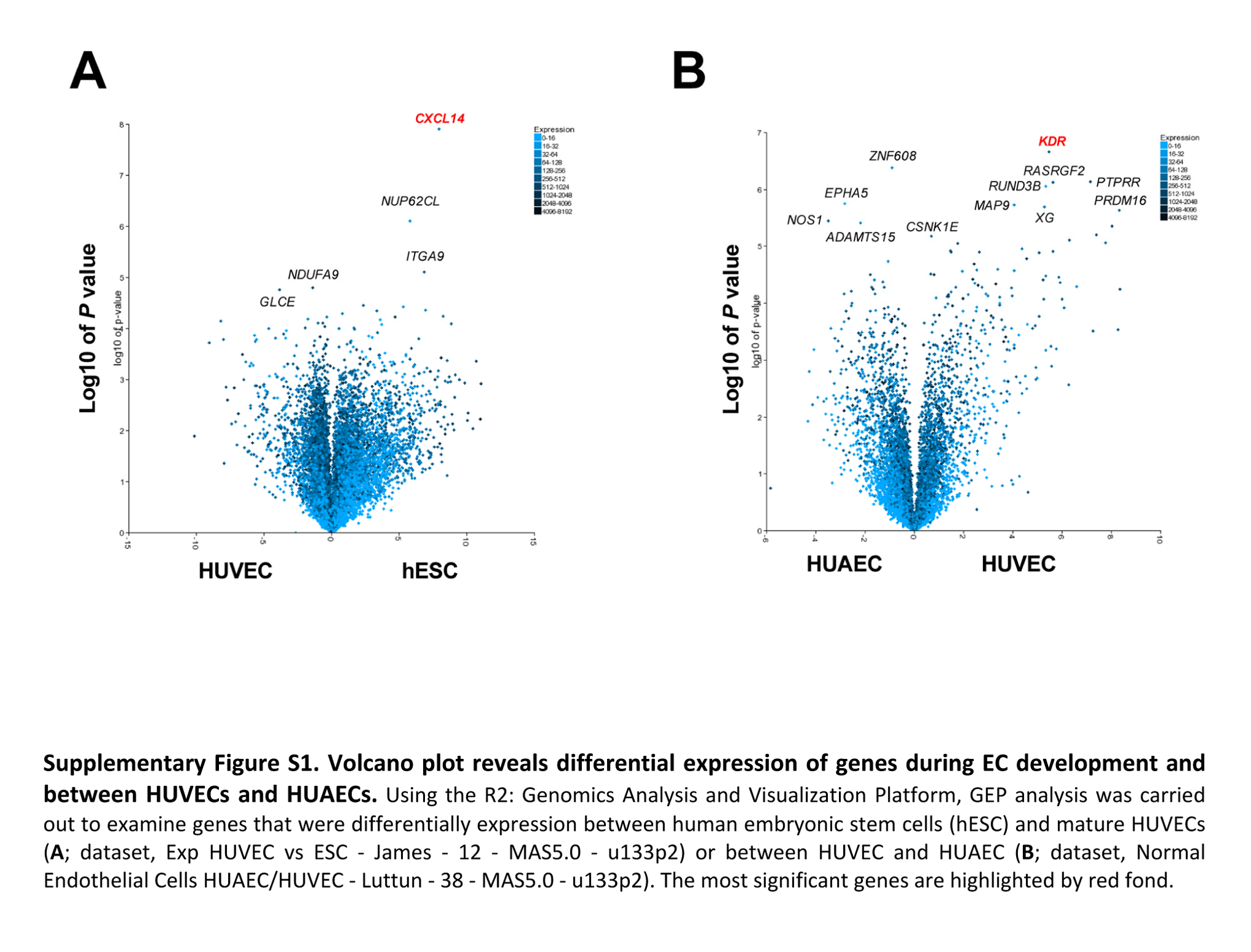

Supplement: Supplementary Figure 1 [file aging-11-101726-s001.tif]

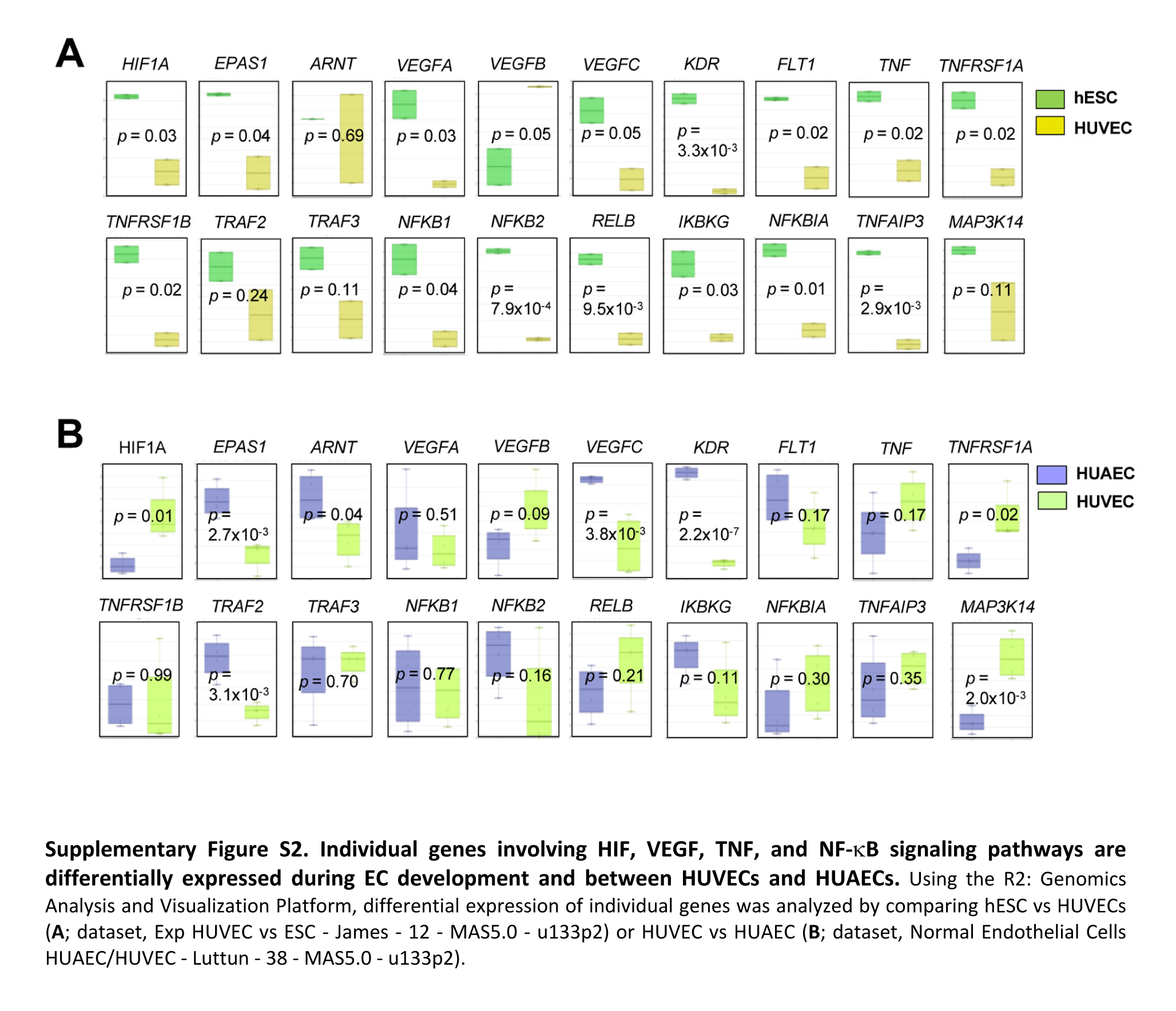

Supplement: Supplementary Figure 2 [file aging-11-101726-s002.tif]

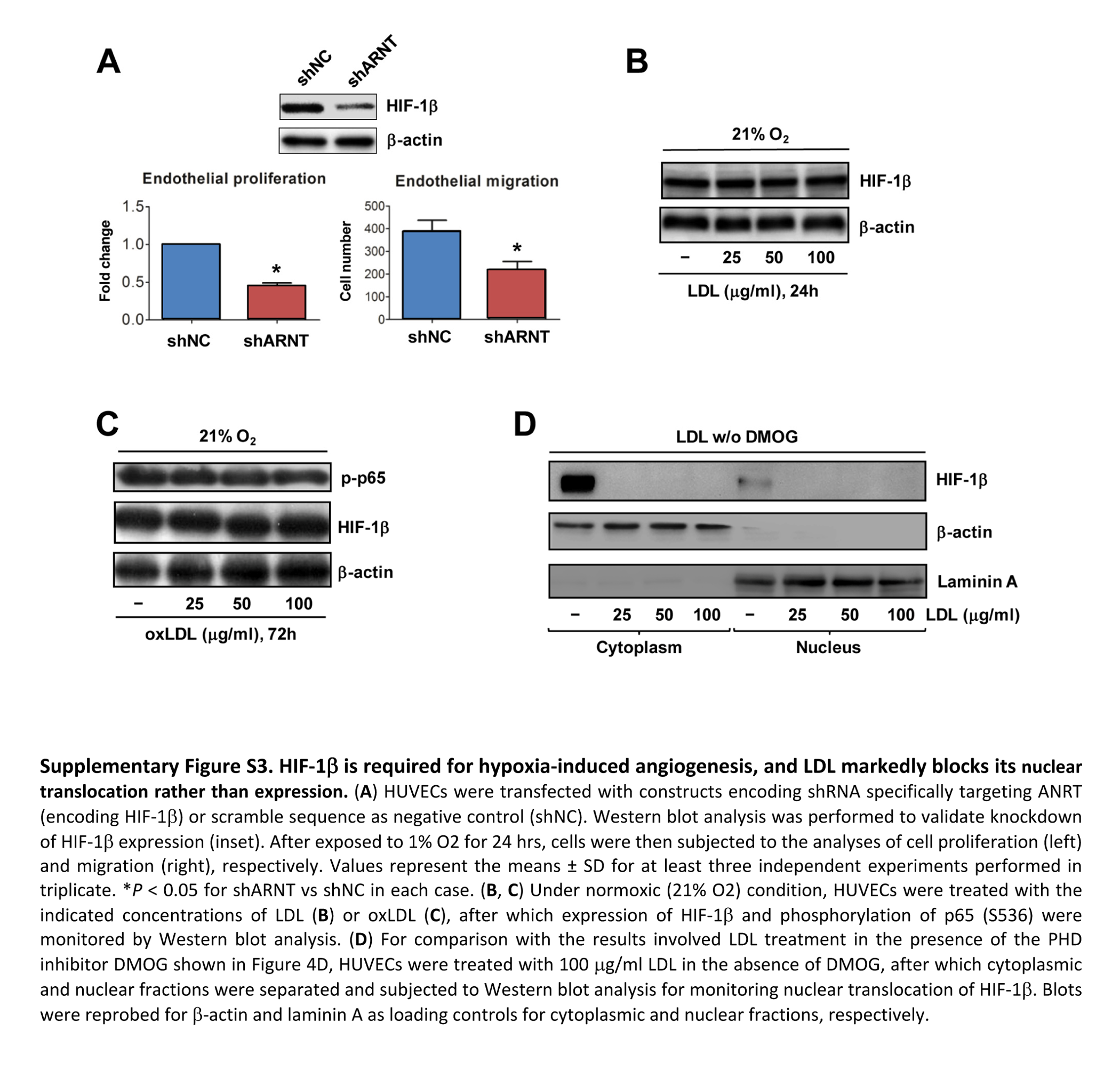

Supplement: Supplementary Figure 3 [file aging-11-101726-s003.tif]

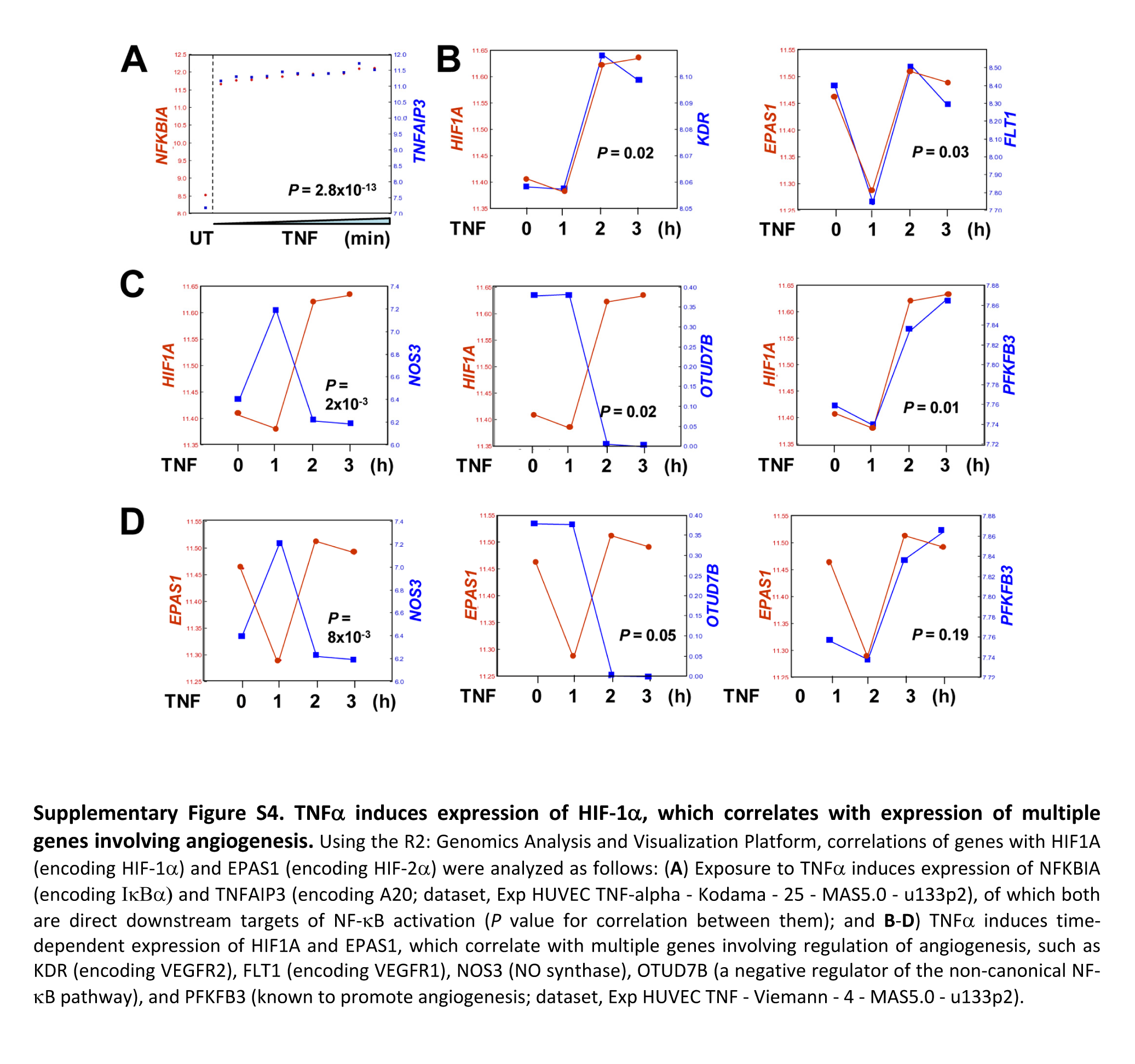

Supplement: Supplementary Figure 4 [file aging-11-101726-s004.tif]

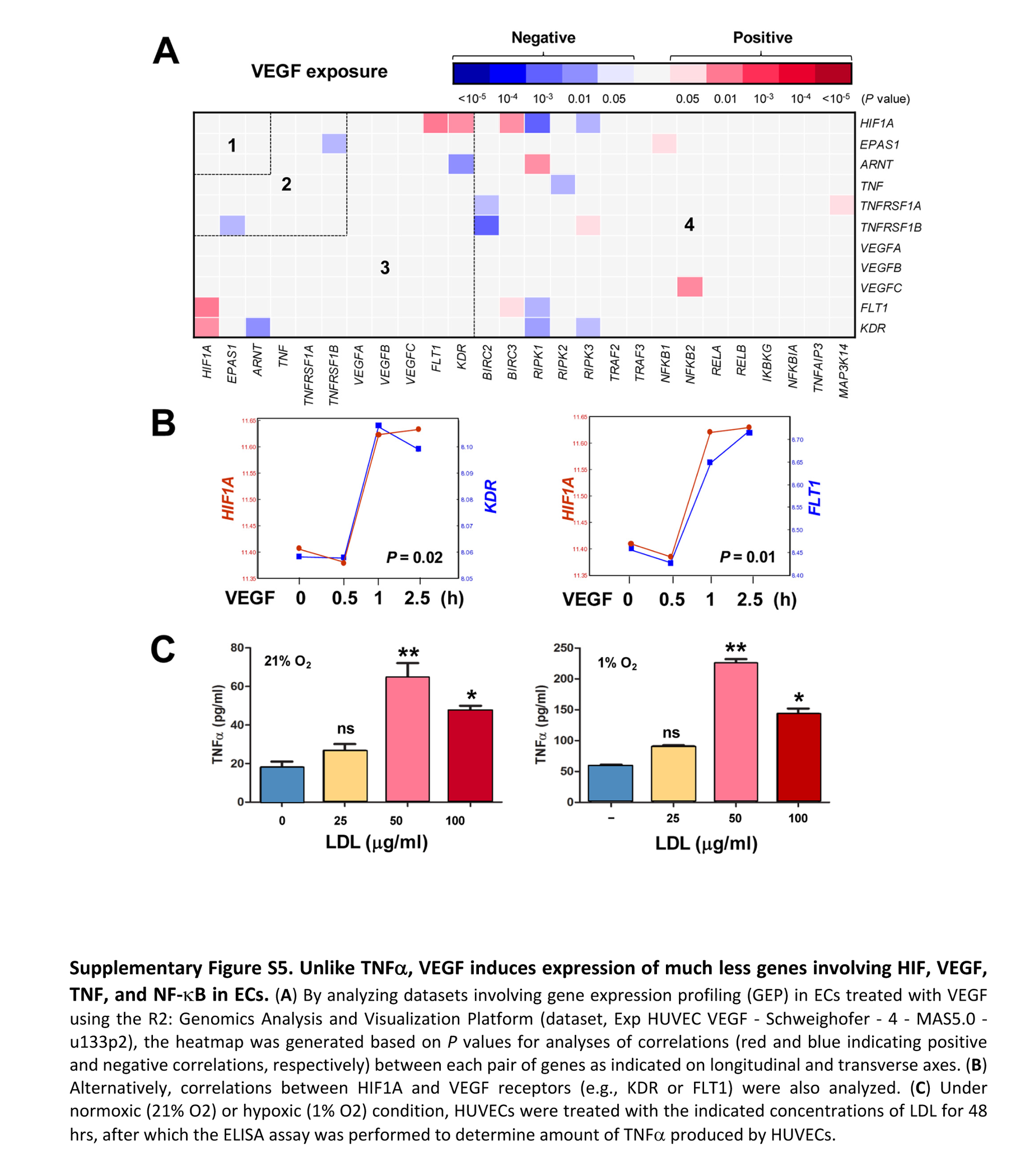

Supplement: Supplementary Figure 5 [file aging-11-101726-s005.tif]
